# OutSplice: A Novel Tool for the Identification of Tumor-Specific Alternative Splicing Events

**DOI:** 10.3390/biomedinformatics3040053

**Published:** 2023-10-08

**Authors:** Joseph Bendik, Sandhya Kalavacherla, Nicholas Webster, Joseph Califano, Elana J. Fertig, Michael F. Ochs, Hannah Carter, Theresa Guo

**Affiliations:** 1 Moores Cancer Center, University of California San Diego, San Diego, CA 92037, USA; 2 Gleiberman Head and Neck Cancer Center, University of California, San Diego, CA 92037, USA; 3 Department of Otolaryngology-Head and Neck Surgery, University of California San Diego, San Diego, CA 92037, USA; 4 Quantitative Sciences Division and Convergence Institute, Sidney Kimmel Comprehensive Cancer Center, Johns Hopkins University, Baltimore, MD 21224, USA; 5 Department of Oncology, Johns Hopkins University, Baltimore, MD 21224, USA; 6 Department of Biomedical Engineering, Johns Hopkins University, Baltimore, MD 21224, USA; 7 Department of Applied Mathematics and Statistics, Johns Hopkins University, Baltimore, MD 21224, USA; 8 Department of Mathematics and Statistics, The College of New Jersey, Ewing, NJ 08628, USA; 9 Division of Medical Genetics, Department of Medicine, University of California San Diego, San Diego, CA 92093, USA

**Keywords:** algorithm, benchmarking, alternative splicing, head and neck cancer, software

## Abstract

Protein variation that occurs during alternative splicing has been shown to play a major role in disease onset and oncogenesis. Due to this, we have developed OutSplice, a user-friendly algorithm to classify splicing outliers in tumor samples compared to a distribution of normal samples. Several tools have previously been developed to help uncover splicing events, each coming with varying methodologies, complexities, and features that can make it difficult for a new researcher to use or to determine which tool they should be using. Therefore, we benchmarked several algorithms to determine which may be best for a particular user’s needs and demonstrate how OutSplice differs from these methodologies. We find that despite detecting a lower number of genes with significant aberrant events, OutSplice is able to identify those that are biologically impactful. Additionally, we identify 17 genes that contain significant splicing alterations in tumor tissue that were discovered across at least 5 of the tested algorithms, making them good candidates for future studies. Overall, researchers should consider a combined use of OutSplice with other splicing software to help provide additional validation for aberrant splicing events and to narrow down biologically relevant events.

## 1. Introduction

Alternative splicing is a crucial biological process involving the differential inclusion, exclusion, and rearrangement of exons across eukaryotic tissues. This results in coding regions from the same gene being stitched together in various possible arrangements, allowing for a vast expansion of protein diversity as well as alterations in protein phenotype and expression [1]. The variation that occurs during this post-transcriptional process presents a significant source of functional changes in proteins and allows for the onset of numerous diseases, including cancer [2]. Many studies have demonstrated that aberrant splicing plays a major role in the oncogenesis of several cancers. For example, CD44 (cell-surface receptor) has numerous splice variants whose up-regulation contributes to the onset and metastasis of breast and colon cancer [3–5]. Additionally, exon skipping events on MDM2 (proto-oncogene) produce the MDM2-ALT1 alternative isoform, whose expression causes accelerated tumorigenesis in rhabdomyosarcomas [6].

Determining the impact of splicing on cancer requires not only identifying each variant but also further characterizing whether this event is tumor-specific. In the case of head and neck cancer, we have previously shown that splice events can cause mutation-independent oncogenic pathway activation [7]. The ability of aberrant splicing to change protein function specific to tumors also results in the generation of neoantigens that can represent novel targets for enhancing response to immunotherapy [8–10]. While the study of somatic mutations alone has led to the discovery of neoantigens in cancers, these alterations may be limited in tumors with a low mutational burden [11,12]. This is particularly evident in head and neck cancer, whose tumors display variable mutational changes but have a large degree of splicing events [8,13]. In this context, knowledge of tumor-specific alternative splicing events is critical to our understanding of head and neck cancer onset and to the creation of drugs for immunotherapy treatments.

Previously, we developed an algorithm to detect splicing based on the outlier expression of junctions in tumors relative to normal samples [7]. Multiple methods and software packages have been developed in the past for documenting alternative splicing. This includes transcript isoform estimation [14–17], differential exon expression [18–21], the generation of inclusion levels [22–26], and building splicing graphs [27–31]. While many of these methods are powerful, they are often more complex than our outlier junction approach and capture differences in mean isoform usage that are not tailored for handling heterogeneous tumor transcriptomes [32]. Therefore, we have developed OutSplice, a software package in Bioconductor with a GenePattern implementation for our outlier analytics to identify novel splicing events in a user-friendly fashion and determine splicing events as they occur in each individual tumor [7,33,34]. Given all the methods for differential splicing analysis, it is crucial to determine which algorithms and software will be best for each individual user’s needs. Previous studies have looked into comparing different splicing tools; however, these studies primarily focus on benchmarking measures such as precision, recall, and FDR [35,36]. While this is crucial to tool selection, it is also important to compare the complete pipelines of these tools, as they each have varying complexities in their usage and differences in their output. This “ease-of-use” may be helpful for new researchers. This includes identifying tools requiring fewer processing steps and scripting knowledge to ease entry into the field.

We therefore compared OutSplice to several differential splicing detection algorithms (edgeR, LeafCutter, psichomics, rMATS, and Whippet) by benchmarking the number of significant events found, runtime, required memory, required inputs and outputs, and general usability against an RNA-seq dataset of 47 oropharyngeal squamous cell carcinoma tumors (OPSCC) and 25 normal samples. A synthetic RNA-seq dataset was also created to measure each model’s performance against “ground truth” over-expressed alternatively spliced transcripts. Each algorithm used here was selected to represent every major splicing methodology. Transcript isoform estimation, however, was not included in this study as the current primary software utilizing this method, cuffdiff and MISO, demonstrate either extremely long runtimes or only utilize pairwise sample comparisons. Instead, given the similarities between psichomics and OutSplice regarding how junction read counts are measured, this tool was also selected along with the popular rMATS software. Additionally, we also compared OutSplice to other previously developed outlier detection methods, FRASER and LeafCutterMD [37,38]. Here we describe how OutSplice is unique from these previously developed tools and identify 17 genes that matched across at least 5 of the tested algorithms, providing high confidence for the presence of biologically relevant aberrant splicing.

## 2. Materials and Methods

### 2.1. Data Preparation

The 47 primary tumor tissue samples used for the differential splicing analyses in each algorithm were obtained from a patient cohort with HPV-related OPSCC [39]. The 25 normal tissue samples used for the comparisons consisted of oropharynx mucosal tissue obtained from uvulopalatopharyngoplasty (UPPP) surgical samples in cancer-unaffected controls. RNA was extracted using the mirVana miRNA Isolation Kit (Ambio, Forster City, CA, USA) and quantified with a NanoDrop spectrophotometer (Thermo Fisher Scientific, Waltham, MA, USA). The Illumina TruSeq stranded total RNA seq poly A+ Gold reduction kit was used to prepare the RNA library (San Diego, CA, USA). RNA was then sequenced using the HiSeq 2500 platform sequencer (Illumina, San Diego, CA, USA) with the TruSeq Cluster Kit (Illumina, San Diego, CA, USA), resulting in a mean read depth of 80 million 100 × 100 paired-end reads per sample that were then trimmed to remove adaper sequences and low-quality reads. Final compressed FASTQ files ranged from sizes of 1.4 gigabytes (GB) to 6.6 GB.

### 2.2. Computational Resources

Every algorithm and software were run on the National Resource for Network Biology (NRNB) cluster hosted by the San Diego Supercomputer Center (SDSC) using the Simple Linux Utility for Resource Management (SLURM) system for job management and measuring resource usage. Every step in the pipeline for each algorithm was given a maximum allocation of 8 CPUs. Elapsed time and max resident set size (maxRSS) were recorded for each step in the corresponding algorithm pipeline. For pipeline steps that required samples to be processed individually, an array job was submitted to run in parallel, with runtime being recorded as an average across all samples.

### 2.3. Genome Index Building and Alignment

For the algorithms that required Binary Alignment Map (BAM) files containing aligned reads, genome indices were built and aligned using STAR v2.7.1a [40]. The reference annotation and assembly files required to build the reference were obtained from GENCODE [41]. All reads present in the trimmed FASTQ files were decompressed and aligned to the Human Genome Build 38 (hg38). STAR 2 Pass Mode was used to improve splice junction quantification [42].

### 2.4. Algorithm Data Formatting Pipelines

All algorithm pipelines were run by following the usage guides for each corresponding step in their pipeline, starting from FASTQ file input, to emulate the process an average user would need to use after getting their sequencing data. Pipelines were run using the default settings with the following exceptions made when available: library type (upstream reads were derived from the reverse strand), # of threads (8), paired end reads (True). The significance threshold was set to a false discovery rate (FDR) adjusted *p*-value of 0.05 for all algorithms. All splicing events were mapped to the hg38 genome for annotation.

#### 2.4.1. OutSplice Pipeline

For OutSplice, junction read counts were obtained from STAR’s SJ.out.tab output. The total number of raw counts was obtained from STAR’s #Uniquely Mapped Reads result in the Log.Final.out output. Gene expression data for OutSplice was obtained by following the Cancer Genome Atlas (TCGA) RSEM v2 normalization pipeline using RSEM v1.3.1 [15,43]. The RSEM transcript reference directory was created using the aforementioned annotation and assembly files from GENCODE.

To help users prepare and format their data for use with OutSplice, we have developed the OutSplice Formatter tool, which will extract junction count, raw count, and gene expression data from STAR and RSEM output for use with the actual algorithm. Utilizing a perl script from the University of North Carolina’s Bioinformatics utilities, an optional upper quartile normalization is integrated into the Formatter and was used to normalize the expected gene counts from RSEM (https://github.com/mozack/ubu, accessed on 16 March 2023). This formatter is open source and available on our GitHub (https://github.com/GuoLabUCSD/OutSpliceFormatter/tree/Version1.0.0, accessed on 16 March 2023).

When running OutSplice v1.0.1, the program will first convert the raw junction counts from STAR into reads per million (RPM) by dividing the original counts by the total number of raw counts. These are then further normalized by gene expression by dividing by the quartile normalized expression values from RSEM. Outliers are then detected through a rank sum approach, a modified version of the Ghosh method, where an outlier must have greater than 0.00001 normalized RPM relative to gene expression [44,45]. OutSplice is open source and available on Bioconductor and GenePattern (https://bioconductor.org/packages/release/bioc/html/OutSplice.html, accessed on 26 April 2023).

#### 2.4.2. edgeR Pipeline

Differential splicing was performed using edgeR v3.42.2. For edgeR, read counts were obtained using the featureCounts function in Rsubread v2.14.2 [46,47]. Default settings were used with exceptions for paired end reads, exclusion of meta features, and multiple overlap as per the edgeR user guide for alternative splicing analysis (https://bioconductor.org/packages/release/bioc/vignettes/edgeR/inst/doc/edgeRUsersGuide.pdf, accessed on 16 March 2023). Genes were filtered to only include those with at least 10 read counts in a minimum number of samples and 15 counts across all samples. Normalization of the data was then performed using the recommended and default trimmed mean of M values (TMM) method.

#### 2.4.3. LeafCutter/LeafCutterMD Pipeline

Aligned BAM files generated by STAR were indexed using SAMtools v1.15.1 and converted to JUNC files using RegTools v.0.5.2 [48,49] with an anchor length of 8 base pairs (bp) and a required intron length between 70 and 500,000 bp. A Python script provided by LeafCutter v0.2.9 was then run to perform intron clustering, where 30 split reads were required to support the identification of a cluster to be passed into the LeafCutter differential splicing script. Annotation codes were made using the beforementioned hg38 annotation file from GENCODE and given to LeafCutter’s visualization script for use with the LeafViz application. For LeafCutterMD, the above steps were repeated on 25 tumor samples for comparison with FRASER.

#### 2.4.4. Psichomics Pipeline

Differential splicing was performed using psichomics v1.26.0. (https://nuno-agostinho.github.io/psichomics/articles/custom_data.html, https://nuno-agostinho.github.io/psichomics/articles/CLI_tutorial.html, accessed on 16 March 2023). Junction counts were obtained from STAR’s SJ.out.tab file. Per the usage guide, events were filtered to include only those with a change in percent spliced in (PSI) greater than 0.1 to help remove non-biologically relevant events.

#### 2.4.5. rMATs Pipeline

Differential splicing was performed using rMATS v4.1.0. STAR-aligned BAM files were used for the initial input. A read length of 100 was specified along with the variable read length option to account for the trimmed reads.

#### 2.4.6. Whippet Pipeline

A Whippet v1.6.1 provided Julia script was first used to quantify the original FASTQ files, and the software-specific index was built using the same annotation and assembly files used for STAR. The resulting PSI files were then passed into Whippet’s “delta” script for differential splicing analyses. The resulting events were then filtered to include only those whose change in PSI was greater than 0.1 and had a splicing probability of at least 90%.

#### 2.4.7. FRASER Pipeline

Outlier analysis was performed using FRASER v1.12.1 on 25 tumor samples. Split and non-spliced reads were extracted from the aligned BAM files using FRASER’s countRNA-Data function. Read counts were then filtered and normalized by requiring 20 split reads and an absolute change in PSI of 0.3 for an intron to pass the filter. Hyperparameters utilized were also tuned and set prior to model fitting (http://www.bioconductor.org/packages/release/bioc/vignettes/FRASER/inst/doc/FRASER.pdf, accessed on 16 March 2023).

#### 2.4.8. Simulated Data Creation

The first 1000 genes present on chromosome 1 were selected for use from the GENCODE hg38 gtf file. Uncharacterized genes on open reading frames, RPS genes, and those listed without a HGNC symbol were ignored. For each gene, the Ensembl Canonical transcript was selected from the gtf file. Additionally, for 200 genes, an alternatively spliced transcript was selected. These data were then provided to Polyester to generate FASTA files containing the simulated reads for 20 “Normals” and 40 “Tumors” using a baseline number of reads per transcript of 600 [50]. To establish a “ground truth” for event over-expression and to simulate tumor heterogenity, transcripts were given varying expression levels relative to the baseline. Canonical transcripts were given an expression level between 1 and 2× the baseline expression. Alternatively, spliced transcripts were then given an expression level of 1× for the normal samples, 1–2× for 20 tumor samples, and 4–5× for the remaining 20. These 200 transcripts would then be considered to have true overexpression for the calculation of the evaluation metrics. BBTools was then used to convert the polyester output into FASTQ format for use with each splicing algorithm (https://sourceforge.net/projects/bbmap/, accessed on 16 May 2022). Each algorithm was run as previously described, with an exception for the unstranded library type. OutSplice was run with a fold-change cutoff of 2 instead of the default value of 10 to account for the overall lower degree of expression variation.

## 3. Results and Discussion

### 3.1. OutSplice

When considering differential splicing in the context of cancer research, the heterogeneity of tumors can make it difficult to identify both the splicing events that occur across the cohort and the individual events that occur per sample. To combat this, OutSplice was specifically designed to work with tumor biology to detect differences in splicing between tumor and normal samples. To do such, OutSplice integrates a software package to implement a modified version of the Ghosh method to call outlier splicing events by setting a minimum level of expression relative to normal samples [44,45]. In this algorithm, each tumor sample’s normalized junction expression is compared to the distribution of normal samples, allowing for the identification of an outlier event per sample. Next, a Fisher’s exact test with an FDR correction is performed on each event, comparing the number of outliers in each group, so both over- and under-expressed events occurring in a significant number of tumors will be identified and counted. OutSplice can then determine the splice burden and the types of events that occurred in each sample, allowing users to quickly distinguish which samples in their cohort underwent the highest amount of significant splicing and what kind of events were included. This method has previously been introduced and shown to identify functionally active splice variants of AKT3, DOCK5, and LOXL2 that promote oncogenesis in head and neck cancer [7,33,34]. The development of a unified software package for this outlier analysis in OutSplice and a user-friendly interface with GenePattern notebook facilitates broader applicability for differential splicing analysis in cancer.

**Figure 1.**
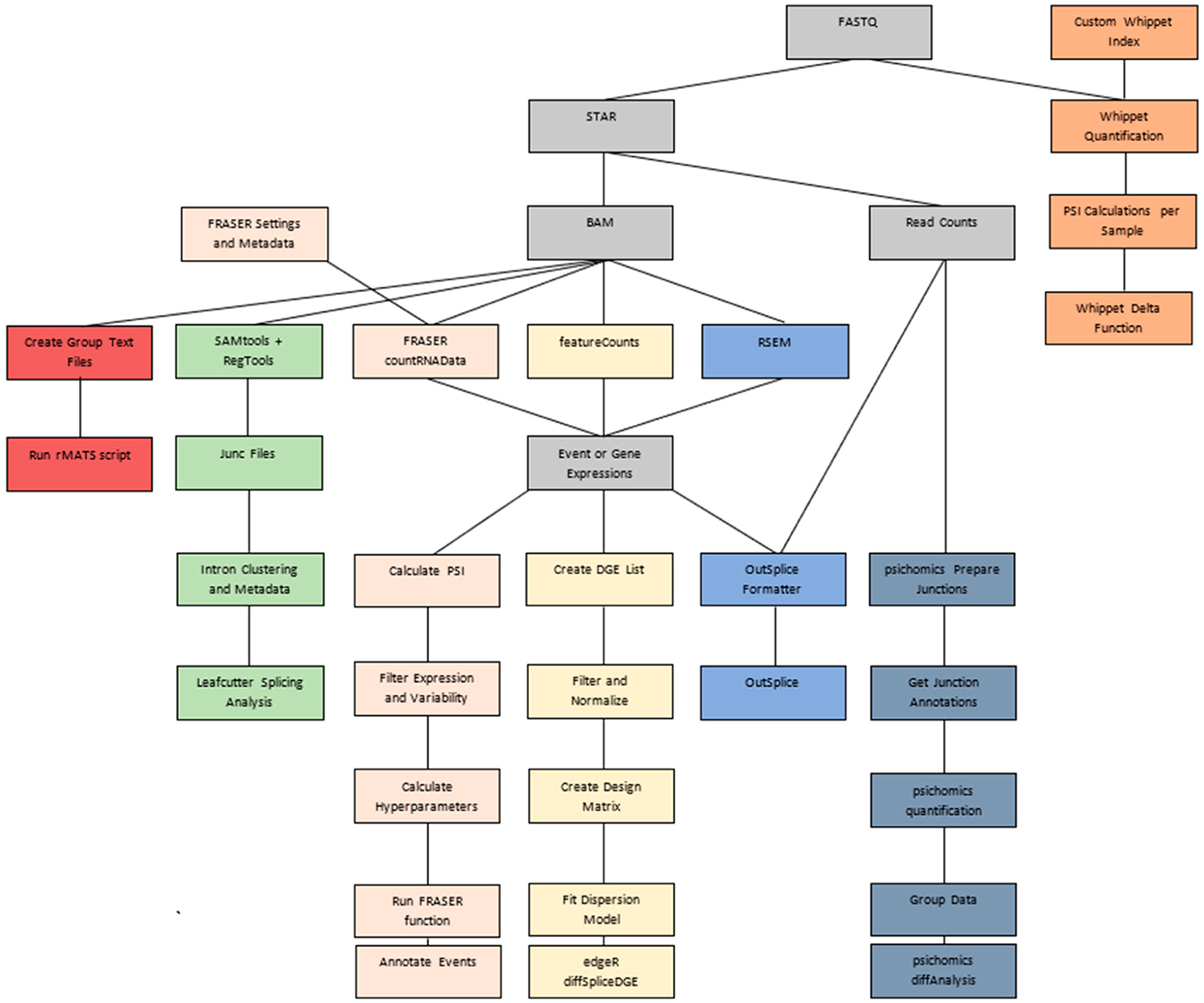
Algorithm pipeline comparisons starting with FASTQ file input. Gray = Input/Output/Tools required by more than one algorithm; Orange = Whippet; Green = LeafCutter; Pink = FRASER; Yellow = edgeR; Blue = OutSplice; Aegean = psichomics; and Red = rMATS.

Among the algorithms for differential splicing, OutSplice is among those that require the fewest processing steps (Figure 1, Supplementary Table S1). The formatter and algorithm are both able to be run in one function, so users will not have to do their own separate normalizations or data handling. Additionally, it contains a function that is formatted to work immediately with data downloaded from the TCGA/Firebrowse. Firebrowse is a collection of processed data from the TCGA for numerous cancer types and contains junction and gene expression data. This direct linkage to TCGA analysis could be of great benefit to researchers who would like to study splicing in various types of cancer.

When benchmarking the runtime and memory requirements for OutSplice compared to other differential splicing tools, OutSplice was among the algorithms with higher memory usage and longer overall runtimes (Supplementary Table S2). A major reason for this is that external software, STAR and RSEM, were used to get the expression counts for the algorithm, lengthening the process. Here, STAR’s SJ.out.tab output is used to provide the number of uniquely mapping reads that specifically span exon-exon junctions instead of all possible overlapping reads that algorithms such as edgeR utilize. This may be beneficial as it can better designate the specific splicing event that is occurring rather than just the overall expression of an exon. Optionally, RSEM can then be used on a per-sample basis to determine the gene expression level that these junctions will be normalized by. While the direct normalization of STAR’s gene count output would be faster overall, RSEM may be more effective as a measure of gene expression given its ability to better handle ambiguous and multimapping reads [15].

### 3.2. edgeR

EdgeR detects splicing alterations by measuring the difference in exon expression between groups based on the log fold change in exon expression compared to the log fold change of gene expression [18]. To determine expression levels in each exon, edgeR fits a negative binomial model based on a matrix of read counts provided by external software such as STAR and featureCounts. These tools will measure all reads that align with each exon in the sample to provide a representation of the expression level of each coding region.

When starting from raw FASTQ input, the overall edgeR pipeline was found to have one of the longest runtimes and the highest amount of data formatting compared to the other available software. The biggest contributor to this length was FeatureCounts, which took the longest time to process all 72 samples and generate a count matrix. Disregarding alignment and read counting, this tool does demonstrate one of the fastest runtimes; however, there are several steps involved in the preparation process that must be written in an R script. While user guides can help, new users may struggle to deal with specifics in their data. For example, standard GENCODE gtf files will list paralogous genes and ENSEMBL version numbers that cause edgeR to throw an error due to duplicate and non-standard row names. Therefore, this method will require more time based on user knowledge compared to other tools.

EdgeR does offer many benefits to users. EdgeR is available as a Bioconductor package, making installation fairly straightforward, and it offers the largest variety of options regarding data processing compared to other algorithms. This can be of great use to researchers who want to use different normalization methods, have more complicated experimental set-ups, or have larger groups. EdgeR’s output is also extremely straightforward to understand, consisting of a single matrix with all exons found and an automatically calculated FDR-corrected *p*-value to help ease significant event identification.

### 3.3. LeafCutter

To identify differences in splicing, LeafCutter focuses on the excision of introns based on clusters of overlapping split reads spanning exon-exon junctions rather than exon expression. This way, splice junctions can be identified without relying on often incomplete, predefined events [22]. From here, LeafCutter then uses a Dirichlet-multinomial model to fit and compare the counts of identified introns in the clusters.

Disregarding read alignment and counting, LeafCutter did take slightly longer than the rest of the tested algorithms to process the data and also required some manual formatting steps such as BAM file indexing with SAMtools and a BAM to JUNC file conversion with RegTools. These steps are able to finish fairly quickly but would most likely warrant the need for a cluster that can process multiple samples at one time if given large datasets and the need to learn other Bioinformatic tools. However, all of these steps will manage read-counting for the user, meaning external software is not required for expression measurement. Further, each of these steps is performed with low memory requirements. If users already have BAM files available, then LeafCutter can make for a good choice for users with few computational resources in exchange for a longer overall runtime.

After generating results, LeafCutter will output FDR-corrected *p*-values at the cluster level for each gene, PSI metrics for each individual event, and, if given a GTF annotation file, canonical/cryptic junction labels. FDR correction at the cluster level could be a potential downside for some users, as this may make the interpretation of significance at individual events difficult. Additionally, if the user would like to map these events to a gene, they will need to manually build annotation codes from a gtf file using the provided function. Despite this, the inclusion of a PSI metric at the event level may be useful for determining the likelihood an individual event will be called, and canonical junction labels greatly aid with the discovery of novel junctions.

### 3.4. Psichomics

When detecting splicing differences between groups, like LeafCutter, psichomics also leverages the number of reads spanning exon-exon junctions to generate inclusion levels. However, instead of using clusters of overlapping introns distinguished by split reads, psichomics calculates the percent spliced in metric for every event in every sample by the proportion of reads aligned to the junction supporting the inclusion isoform [24].

Psichomics includes some major benefits regarding data preparation. While the software does have multiple steps required for processing that require some knowledge of R to manually run each function from the command line, it comes with a graphical user interface that greatly speeds up and eases this process, making it a great resource for those with no programming knowledge. Additionally, like OutSplice, psichomics is also able to work immediately with data from the TCGA/Firebrowse, allowing for instant analysis of cancer-related studies.

After data analysis, psichomics was found to have the lowest memory usage and one of the fastest runtimes across all algorithms. The tool avoids the need to run other software to generate the read matrices and will directly pull the junction mapping results straight from the STAR SJ.out.tab output after alignment and normalize the results for each sample during the PSI calculation. However, it is important to note that if the user does not already have junction read count matrices to quantify, then STAR will be required, raising memory usage.

Some major benefits of psichomics also include their variety of output. FDR-corrected *p*-values are provided for event identification in addition to the PSI calculations and event type. Additionally, psichomics contains in-built principal component analyses, allowing the user to see which events are causing the greatest degree of separation between the two groups in question. However, one major downside of the output is that it relies on the existence of alternative splicing annotations from other tools, such as VAST-TOOLS [51]. While these annotations for hg38 are provided by default, this makes the identification and discovery of novel splicing events difficult [24].

### 3.5. rMATS

Similar to the beforementioned LeafCutter and psichomics packages, rMATS generates inclusion levels based on the number of read counts spanning the splice junctions. However, it also additionally leverages the read counts of the individual exons involved in the event to aid with event identification. To do this, rMATS uses a hierarchial framework with a binomial and logit-normal distribution, allowing the model to account for both estimation uncertainty and replicate variability [26].

The biggest benefit seen with the rMATS pipeline is the few steps required to use the algorithm. Following installation, users just need to create text files designating the experimental and control groups and then run a single all-in-one function. However, rMATS was shown to have one of the longest runtimes as well as the highest memory usage, meaning more computational resources will need to be dedicated for use with this algorithm.

Regarding the resulting output, rMATS provides several useful statistics for the user. This includes an automated annotation of the events with their gene symbol, an FDR-corrected *p*-value, the difference in inclusion levels between the groups tested, and the type of event found.

### 3.6. Whippet

Whippet’s method for differential splicing relies on building splicing graph representations of each sample’s transcriptome [27]. After creating an index from the provided GTF annotations and the genome sequence FASTA, Whippet aligns raw RNA-sequencing reads to a contiguous splicing graph, allowing the user to directly use their FASTQ files with the tool instead of having to install and use an aligner such as STAR.

The first major benefit of Whippet is that every other algorithm tested required a BAM file to run, except for Whippet. This is a major benefit to researchers who do not have access to the high computational power/memory required by RNA-seq aligners (Supplementary Table S2). Whippet is also able to optionally take aligned BAM files to help build the initial index. If the user has experience with merging BAM files, this could be a useful feature to help with the identification of unannotated splicing regions.

Regarding Whippet’s output, the software is able to provide the types of events found and the PSI metric; however, it does not calculate significant *p*-values. Instead of these designations, Whippet suggests that events should be filtered to include those with a Probability Score of at least 90% and a change in PSI greater than 0.1, with adjustments based on user preference and experimental set-up. Whippet also includes entropy metrics that demonstrate the degree of uncertainty associated with the splicing event. Each of these metrics, while beneficial, may be difficult for an inexperienced user to select and interpret properly, so researchers may want to consider their experience level before use. Another potential downside to consider is that Whippet does not automatically convert provided Ensembl IDs to gene symbols, which means the user will need to be able to use available mapping algorithms such as AnnotationDBI or biomaRt.

### 3.7. Gene Overlap and Algorithm Comparisons

First, we sought to compare the results of differential splicing analysis to our outlier-based analysis method. After obtaining the initial results from each differential splicing algorithm, individual events were mapped to the gene they lie on, and the number of unique genes containing specific splicing events was counted and compared to see the degree of overlap. Regarding the total number, LeafCutter and rMATS were able to identify the greatest number of genes (3159 and 3691 genes, respectively) that could be considered to have significant splicing alterations, whereas OutSplice identified the least (261 genes). The first reason for this lower number of identifications is partially because, by default, OutSplice will filter out junctions on the X and Y chromosomes, as cancer is not specific to differences in biological sex. This resulted in 86 LeafCutter-significant genes and 115 in rMATS not being detected by OutSplice at all. Secondly, OutSplice uses an additional filter where a minimum degree of normalized expression relative to normal must be met before an outlier can be called, thus removing many events that would not be biologically relevant. Psichomics and Whippet also identified fewer genes compared to LeafCutter, at 345 and 1021 genes, respectively. Similarly, psichomics and Whippet allow for significant events to be filtered by having to meet a minimum change in PSI, thus lowering the number of detected genes with significant events. Therefore, while a greater number of identifications is useful for finding new/unannotated splicing events, it should not be the only defining metric to consider when interpreting splicing results.

When determining the number of gene overlaps across the algorithms, the greatest number of overlaps occurred between LeafCutter and rMATS and between LeafCutter and edgeR (Supplementary Figure S1) [52]. The overlap between LeafCutter and rMATS can most likely be explained by their similar methodologies of leveraging the number of split-reads spanning splice junctions to generate inclusion levels. The overlap between LeafCutter and edgeR is most likely explained by both identifying large numbers of genes, thus providing the greatest opportunity for overlap. Interestingly, most other pairwise comparisons of the algorithms did not show high overlap, emphasizing how different each algorithm is at designating what constitutes a significant event. Researchers will need to account for this possibility and potentially run multiple algorithms, as a gene of interest missed by one method may be detected by another.

**Figure 2.**
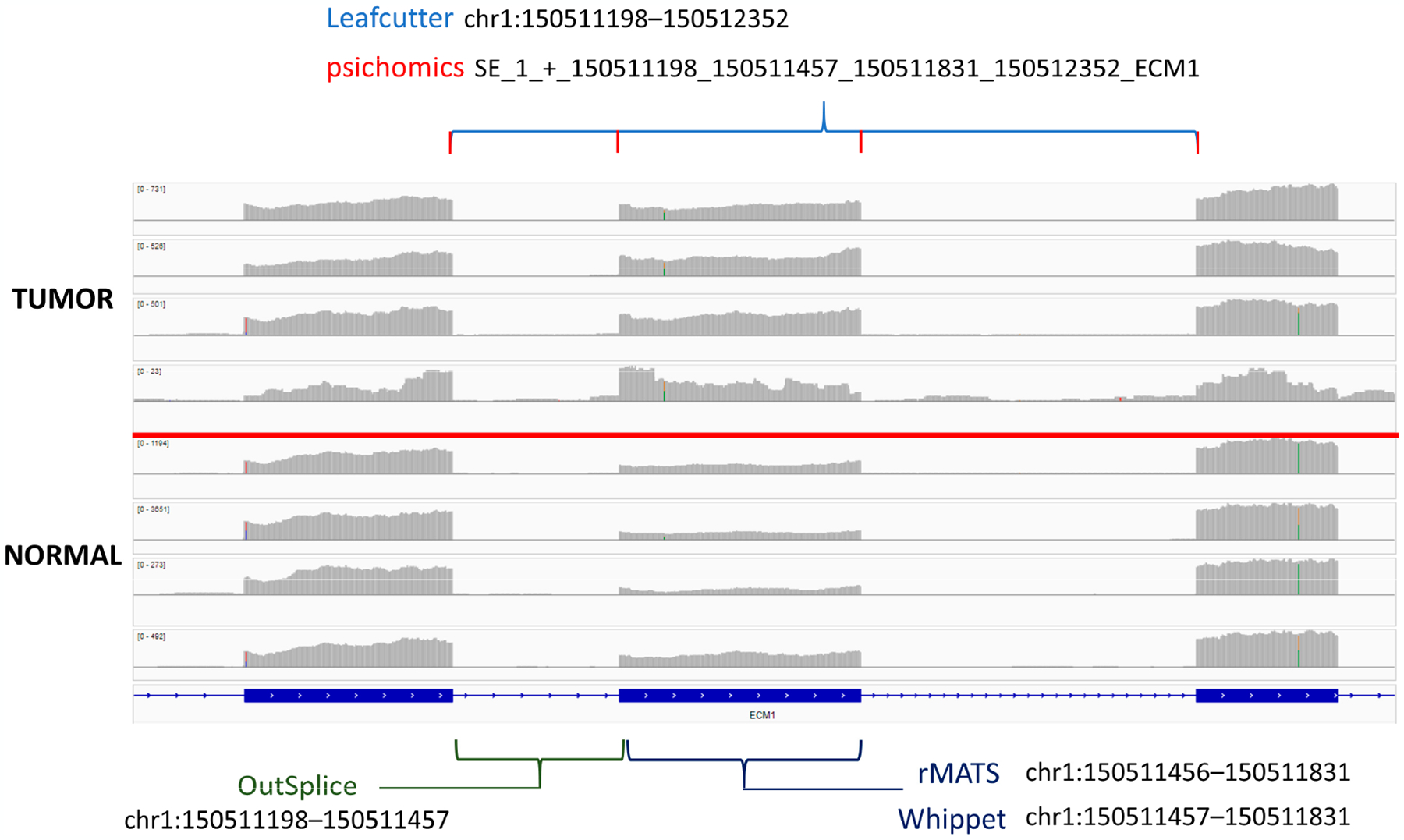
The ECM1 exon “skipping” event was identified by five of the algorithms tested. Brackets indicate the unique coordinate annotation provided by each corresponding algorithm for the singular junction event.

When looking for overlapping genes, 17 genes were found in ≥ 5 of the tested algorithms (Table 1). Multiple algorithms for detecting these provide support for true differential splicing events and could be strong candidates for PCR validation. However, due to differences in coordinate labeling and the large number of potential splice events that can occur on a single gene, it can be difficult to confirm if the exact same splicing event was detected by each tool (Supplementary Table S3). To aid with this task, the interactive genomics viewer can be used to visualize differences in the read alignments across the samples. Through this method, we see that one of these genes, ECM1, displays an easily distinguishable exon skipping event that most algorithms detect exactly but label differently (Figure 2) [53]. Here, the exon at coordinate position chr1:150511457–150511831 shows increased expression in tumor tissue compared to normal. This indicates that the exon is being “skipped” over in normal tissue and may indicate the production of a new peptide in tumors. This figure also illustrates a major challenge in alternative splicing annotation and how there is a lack of a standardized method for event labeling across different algorithms. Researchers will need to take note of this if running multiple different splicing softwares, as different regions that point to the same event could be easily missed. While this event was found to be a major splicing event by most algorithms, it was not marked as significant in edgeR despite the exon being detected. This could be because edgeR’s negative binomial model relies on having low dispersion and low variability across the replicates, which is not usually the case with tumors that are often heterogeneous.

**Figure 3.**
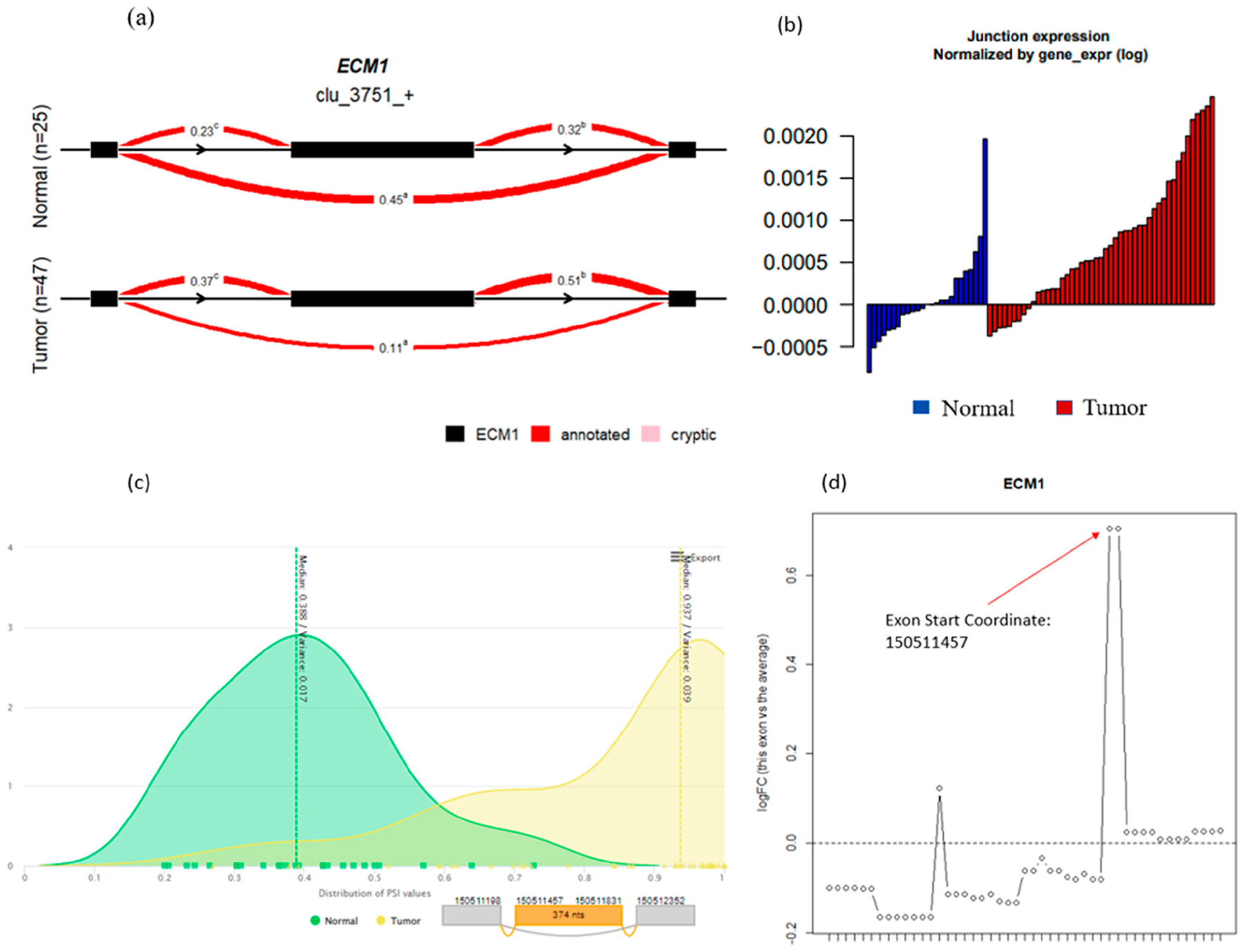
The ECM1 event visualizations were provided, where possible, by various algorithms. (**a**) LeafCutter splicing plot; (**b**) OutSplice normalized junction expression; (**c**) psichomics percent spliced in distributions with splicing plot; (**d**) edgeR log fold changes, where positive changes represent higher exon expression in tumor samples compared to normal.

This ECM1 event can further be viewed through the algorithms themselves. Except for Whippet and rMATS, each algorithm has its own in-built but different method for visualizing significant events that will be up to user preference to decide between when selecting a tool. LeafCutter allows for the installation of LeafViz, which provides nice illustrations of the intron cluster in question and all of the individual splicing events that, when grouped together, are marked as significant. For ECM1, this is noted by the higher PSI for the outer exons compared to the inner “skipped” exon (Figure 3a). OutSplice will provide waterfall plots of the normalized junction expression that allow the user to see how several individual tumor samples overall have a higher expression of the ECM1 event compared to the overall distribution of expression in normal tissue (Figure 3b). Similar to LeafCutter, psichomics also allows you to visualize the ECM1 event’s difference in PSI; however, it does this through distribution plots, which illustrate the higher PSI in tumors compared to normal (Figure 3c). Lastly, edgeR will plot the log fold change in expression of the exon in question. Here, edgeR was able to detect the “skipped” ECM1 exon and showed a positive log fold change, indicating upregulation in tumors; however, it was not able to mark the event as significant (Figure 3d).

To benchmark OutSplice with other available algorithms for differential splicing analysis, we generated in silico RNA-seq data. These data only included annotated transcripts and splicing events, with increased expression of these events in half of the simulated “Tumor” samples. In this simulated model, OutSplice had modest performance compared to the other algorithms, having a lower sensitivity and higher false discovery rate (Table 2). OutSplice was designed to identify highly aberrant outliers within heterogeneous datasets that have greater than 10× the difference in event expression between Tumor and Normal sample groups and also to identify novel, unannotated junctions. The performance of these features was not able to be accurately captured in the simulated Polyester data. However, in real-world data, OutSplice has been successful in identifying several functionally active splicing events that have been validated with polymerase chain reactions and functional assays [7,33,34,54,55].

### 3.8. Outlier Analysis and Comparison

In addition to OutSplice, two primary alternative methods that exist for identifying splicing outliers are LeafCutterMD and FRASER. However, unlike OutSplice, both of these algorithms are intended for the discovery of individual outliers in the context of rare diseases. LeafCutterMD, like regular LeafCutter, uses the same intron clustering and Dirichlet-Multinomial approach to detect splicing events but includes statistical tests to determine how likely the intron counts are coming from the actual distribution [38]. With FRASER, alternative acceptors, donors, and splicing efficiencies are quantified based on the split and non-split reads across exon-exon junctions. The data is then fit with a beta-binomial distribution for each metric, and outliers are found as those that deviate from the model [37].

To compare FRASER to LeafCutterMD, we looked at the same aforementioned benchmarks; however, due to the high memory requirement needed to count split reads with FRASER, the dataset was trimmed to only include 25 of the tumor samples. Here, we found LeafCutterMD to be far less memory-intensive while also having a much faster runtime. After STAR alignment and intron clustering, we found that LeafCutterMD was able to finish the outlier analytics in just 8 min with 4 GB of memory. Due to FRASER’s RNA counting step, the algorithm’s pipeline took significantly longer at approximately 15 h, with a high memory usage of 197 GB, making it more difficult to run for users with minimum computational resources. Additionally, it was found that LeafCutterMD was able to identify more genes with significant splicing outliers at 166, compared to the 33 found with FRASER.

However, there are some important benefits regarding FRASER’s output compared to LeafCutterMD. Unlike FRASER, LeafCutterMD users will need to manually get gene symbol annotations with packages such as GenomicRanges to map the often-large number of provided gene coordinates while also having to manually adjust *p*-values. Additionally, unlike both OutSplice and LeafCutterMD, FRASER can calculate splicing efficiency, which is useful for those studying intron retention.

While LeafCutterMD and FRASER can be used for the detection of splicing outliers, both of these methods are not meant to be used for the comparison of tumor samples and controls included with OutSplice but rather are specifically designed to identify rare events across the disease group only. This means that if many tumors contain an event that is an outlier compared to normal but not to each other, then it will not be detected through these two methods. Since tumors are highly variable with a high degree of splicing alterations compared to normal, it is crucial to identify both significantly shared outliers and significant individual outliers. OutSplice is uniquely capable of performing both differential splicing and outlier splicing, and in doing so, we will be able to determine if there is a particularly common splicing region producing a protein that can be targeted during immunotherapy or if the region is so unique that treatment will only work in some select patients.

## 4. Conclusions

Overall, we find that OutSplice will be most useful for the identification of rare splicing events in line with tumor biology. However, if a user would like to screen for the largest number of potentially significant alterations from all known junction events, LeafCutter may be the best choice. In a setting with low computational resources, Whippet is another option. After running each algorithm, we also found minimal overlap in the discovered splicing alterations across the algorithms. Future studies should include the development of a pipeline capable of running each tool and compiling the results to automatically match genes or specific junction coordinates to provide higher confidence for identified events.

## Supplementary Material

Table S2

Table S1

Figure S1

Table S3

## Figures and Tables

**Table 1.** Genes with significant differential splicing found in ≥5 of the tested differential splicing algorithms, their descriptions, and the types of events found. “X” indicates which algorithms identified significant splicing in the given gene.

Gene with Significant Splicing Event(s)	Description	Event Types Found	edgeR	LeafCutter	OutSplice	Psichomics	rMATS	Whippet
ECM1	Extracellular Matrix Protein 1	Skipping and Alternative 5′ Splice Sites		X	X	X	X	X
COL6A3	Collagen type VI alpha-3 chain	Skipping and Mutually Exclusive Exons	X	X		X	X	X
KIAA1217	Embryonic skeletal system development	Skipping, Alternative First Exon, Retained Intron, and Mutually Exclusive Exons	X	X		X	X	X
HDAC9	Histone deacetylase 9	Skipping, Alternative First Exon, Tandam Start Site, and Mutually Exclusive Exons	X	X		X	X	X
MBNL1	Muscleblind-like splicing regulator 1	Skipping and Mutually Exclusive Exons	X	X		X	X	X
VPS39	VPS39 subunit of the HOPS complex	Skipping and Tandam Start Site	X	X		X	X	X
PLEKHG1	Pleckstrin homology and RhoGEF domain containing G1	Skipping, Insertion, Tandam Start Site, and Mutually Exclusive Exons	X	X	X	X	X	X
ITGB4	Integrin subunit beta 4	Skipping	X	X	X	X	X	X
PTPN6	Protein tyrosine phosphatase non-receptor type 6	Skipping, Alternative Acceptor, Alternative First Exon, Alternative 3′ Start Site, Mutually Exclusive Exons, and Retained Introns	X	X		X	X	X
MTMR1	Myotubularin-related protein 1	Skipping and Mutually Exclusive Exons	X	X		X	X	X
PARD3	Par-3 family cell polarity regulator	Skipping, Alternative 5′ Splice Site, Alternative First Exon, and Mutually Exclusive Exons	X	X		X	X	X
NUMA1	Nuclear mitotic apparatus protein 1	Alternative First Exon, Tandem Transcription Start Site, Retained Intron, and Mutually Exclusive Exons	X	X		X	X	X
RABGAP1L	RAB GTPase-activating protein 1	Skipping and Insertion	X	X	X	X	X	
MDM2	Proto-oncogene	Skipping, Alternative First Exon, and Mutually Exclusive Exons	X	X	X	X	X	
MCM7	Minichromosome maintenance complex component 7	Skipping, Alternative First Exon, and Retained Intron	X	X	X	X	X	
MEI1	Meiotic double-stranded break formation protein 1	Skipping, Insertion, and Deletion Events	X	X	X	X	X	
FCGR2B	FC gamma receptor IIb	Skipping and Retained Intron	X	X	X	X	X	

**Table 2.** Evaluation metrics for each algorithm on the simulated dataset. FDR indicates the False Discovery Rate. “Identified Genes” indicates genes with splicing events that were detected by the algorithm regardless of significance level. “Identified + Significant Genes” indicates genes with splicing events that were both detected by the algorithm and significant at an algorithm-provided *p*-value of ≤0.05. Since Whippet does not record the *p*-value, significance was defined as genes with a splicing probability of at least 90% and a difference in percent spliced value > 0.1.

	Identified Genes					Identified + Significant Genes				
	True Positive	Total Identified	Sensitivity	Specificity	FDR	True Positive	Total Identified + Significant	Sensitivity	Specificity	FDR
edgeR	200	1014	1	0.03	0.8	192	265	0.96	0.91	0.28
LeafCutter	185	213	0.93	0.97	0.13	181	194	0.91	0.98	0.07
OutSplice	91	366	0.46	0.67	0.75	78	336	0.39	0.69	0.77
psichomics	108	123	0.54	0.98	0.12	102	115	0.51	0.98	0.11
rMATS	149	392	0.75	0.70	0.62	115	120	0.58	0.99	0.04
Whippet	200	972	1	0.07	0.79	77	79	0.39	1	0.03

## Data Availability

The data presented in this study are available on request from the corresponding author. The data are not publicly available due to privacy restrictions but is in the process of being added to a public repository. Previous RNA-Seq junction data is available at: https://www.ncbi.nlm.nih.gov/geo/query/acc.cgi?acc=GSE112026 (accessed on 20 March 2018).
