# Supplementary material for: OutSplice: A Novel Tool for the Identification of Tumor-Specific Alternative Splicing Events
Source: BioMedInformatics. Author manuscript; Available in PMC 2025 Apr 15. (PMC11997874; doi:10.3390/biomedinformatics3040053)
Supplement: Table S2 [file NIHMS2066007-supplement-Table_S2.docx]

**Table S2.** Runtime and maxRSS comparisons for differential splicing algorithms. For steps that were run on a per sample basis, the average time needed per sample is included with the total time for all other steps. Runtime values are rounded to the nearest minute. MaxRSS represents the highest amount of memory needed at a given step during the pipeline. * indicates the step varies by user knowledge. “-” indicates a step not required in a particular algorithm.

|  | **Index + Alignment Runtime** | **Index + Alignment maxRSS** | **Get Read Counts Runtime** | **Get Read Counts maxRSS** | **Formatting and Analysis Runtime** | **Formatting and Analysis maxRSS** |
| --- | --- | --- | --- | --- | --- | --- |
| **edgeR** | 1 H 16 Min  +  1 H, 1 Min per Sample | 36.6 GB | 16 H, 44 Min | 3.5 GB | 23 Min* | 8.9 GB |
| **Leafcutter** |  |  | **-** | **-** | 6 Min per Sample  +  56 Min | 4.6 GB |
| **OutSplice** |  |  | 1 Min  +  1H, 31 Min per Sample | 7.4 GB | 45 Min | 11.7 GB |
| **psichomics** |  |  | **-** | **-** | 22 Min | 3.7 GB |
| **rMATS** |  |  | **-** | **-** | 5 H 30 Min | 23.9 GB |
| **Whippet** | 49 Min  +  1 H, 4 Min per Sample | 4.5 GB | **-** | **-** | 27 Min | 3.8 GB |
