# Supplementary material for: OutSplice: A Novel Tool for the Identification of Tumor-Specific Alternative Splicing Events
Source: BioMedInformatics. Author manuscript; Available in PMC 2025 Apr 15. (PMC11997874; doi:10.3390/biomedinformatics3040053)
Supplement: Table S1 [file NIHMS2066007-supplement-Table_S1.docx]

**Table S1.** Comparisons per step in each algorithm’s pipeline. Columns refer to the process used during each step in the differential analysis pipeline. Rows indicate the specific algorithm. All pipelines begin with FASTQ files as inputs. “-” indicates a step not required in a particular algorithm.

|  | **Index Building and Alignment** | **Get Read Counts** | **Formatting** | **Differential Analysis Method** | **Visualization** |
| --- | --- | --- | --- | --- | --- |
| **edgeR** | STAR | featureCounts | 1. Create DGE List  2. Filter and normalize  3. Create a design matrix  4. Fit dispersion model  5. Run diffSpliceDGE | Differential Exon  Expression | Exon LogFoldChanges |
| **FRASER** |  | Rsubread  countRNAData | 1. Edit FRASER settings  2. Create sample tables  3. Calculate PSI  4. Filter expression and variability  5. Calculate hyperparameters  6. Run FRASER function  7. Annotate Events | Outlier Analytics | Volcano Plots |
| **Leafcutter**  **LeafcutterMD** |  | **-** | 1. Index BAM files  2. Convert BAM to JUNC  3. Run intron clustering  4. Create groups file  5. Run differential splicing / outlier splicing | Differential Intron  Exclusion /  Outlier Analytics | Splice Plots |
| **OutSplice** |  | RSEM  SJ.out.tab  Log.final.out | 1. Specify Groups  2. Run OutSplice Formatter  3. Run outSpliceAnalysis | Outlier Analytics | Waterfall Plots of Expression |
| **psichomics** |  | SJ.out.tab | 1. prepareJunctionquant  2. Get junction annotations  3. Quantification function  4. Group data  5. Run diffAnalyses | Differential Exon Inclusion | Percent spliced in Distribution Plots |
| **rMATS** |  | **-** | 1. Create Groups Text File  2. Run rMATs function | Differential Exon Inclusion | **-** |
| **Whippet** | Whippet Index  +  Quantification Function | **-** | 1. Specify groups  2. Run delta function | PSI Splice Graph  Comparisons | Interactive Genomics Viewer |
