# Supplementary material for: OutSplice: A Novel Tool for the Identification of Tumor-Specific Alternative Splicing Events
Source: BioMedInformatics. Author manuscript; Available in PMC 2025 Apr 15. (PMC11997874; doi:10.3390/biomedinformatics3040053)
Supplement: Figure S1 [file NIHMS2066007-supplement-Figure_S1.pdf]

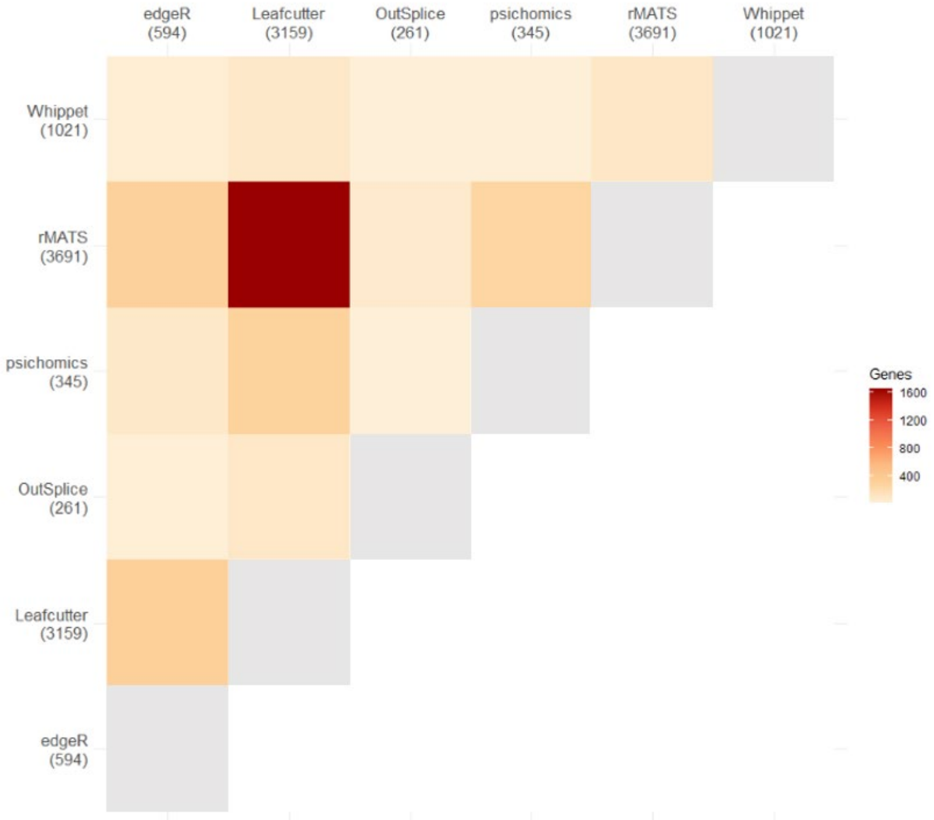

Figure S1. Number of overlapping genes with significant splicing differences between tumor and normal tissue across each algorithm. The number indicates the total number of genes containing significant splicing alterations found by that algorithm.
