# Supplementary material for: OutSplice: A Novel Tool for the Identification of Tumor-Specific Alternative Splicing Events
Source: BioMedInformatics. Author manuscript; Available in PMC 2025 Apr 15. (PMC11997874; doi:10.3390/biomedinformatics3040053)
Supplement: Table S3 [file NIHMS2066007-supplement-Table_S3.docx]

**Table S3:** Significant genomic coordinates provided by each algorithm for each gene with high algorithm overlap. Blank cells indicate that significant events were not found at that gene. *Upstream and Downstream exon coordinates are not listed for rMATS.

| **Gene with Significant Splicing Event(s)** | **Chromosome** | **edgeR**  **Coordinates** | **Leafcutter**  **Coordinates** | **OutSplice**  **Coordinates** | **psichomics**  **Coordinates** | **rMATS** | **Whippet**  **Coordinates** |
| --- | --- | --- | --- | --- | --- | --- | --- |
| ECM1 | 1 |  | 150511198-150512352 | 150511198-150511457 | SE_1_+_150511198_150511457_150511831_150512352_ECM1  A5SS_1_+_150511198_150511831_150512352_ECM1 | 150511456-150511831 | 150511457-150511831 |
| COL6A3 | 2 | 237378636-237379235 | 237377344-237396727  237396847-237425219 |  | SE_2_-_237380915_237379235_237378636_237377344_COL6A3 | 237378635- 237379235  237387581- 237388184  237380914- 237381499  237394586-237395204 | 237378636-237379235 |
| KIAA1217 | 10 | 24466531-24466790  24449433-24449764  24494500-24494604 | 24474060-24495147  24438479-24473228  24533237-24544981 |  | SE_10_+_24474060_24494500_24494604_24495147_KIAA1217  AFE_10_+_24255596_24239337_24380869_KIAA1217 | 24494499-24494604  24536773-24536893  24544980-24547843  24473227-24474060  24495146-24495196  24501378-24501545  24542692-24542770  24542882-24544481 | 24494500-24494604 |
| HDAC9 | 7 | 18509313-18509446  18509273-18509446 | 18496324-18585281  18495854-18496262 |  | AFE_7_+_18509446_18496324_18585281_HDAC9 | 18543168-18543674  18502580-18502639 | 18086949-18087023 |
| MBNL1 | 3 | 152244321-152244440  152446704-152446757  152243828-152244040 | 152445539-152447725  152244440-152414941  152447773-152459271 |  | SE_3_+_152445539_152446704_152446757_152447620_MBNL1  SE_3_+_152447773_152455542_152455577_152456267_MBNL1 | 152299404-152300367  152335110-152335230  152446703-152446757  152455541-152455577  152456266-152456361  152432716-152432920  152447619-152447773  152447724-152447773  152459270-152459345 | 152446704-152446757 |
| VPS39 | 15 | 42192066-42192098 | 42191560-42199896 |  | SE_15_-_42199896_42192098_42192066_42191560_VPS39 | 42192065-42192098 | 42162558-42162672  42162482-42162557 |
| PLEKHG1 | 6 | 150632943-150633304 | 150650786-150733584  150600017-150650728 | 150721286-150733584 | SE_6_+_150600017_150638080_150638125_150650728_PLEKHG1 | 150638079-150638125  150650727-150650786  150733583-150734092 | 150599883-150599884 |
| ITGB4 | 17 | 75755055-75755213 | 75754815-75756429  75737444-75739880  75724782-75727195 | 75757310-75757416 | SE_17_+_75754815_75755055_75755213_75755701_ITGB4 | 75739671- 75739705  75755054- 75755213 | 75755055-75755213 |
| PTPN6 | 12 | 6946610-6946739  6946574-6946739  6946577-6946739  6946710-6946739  6946468-6946739 | 6946739-6952113 |  | SE_12_+_6946739_6951464_6951520_6951609_PTPN6  AFE_12_+_6951520_6946739_6951609_PTPN6 | 6951458-6951520  6951463-6951520  6951608-6952177  6951608-6951731  6951911-6952177  6951982-6952177  6952016-6952177  6951608-6951731 | 6951459-6951463 |
| MTMR1 | X | 150712342-150712365 | 150699300-150727215 |  | SE_X_+_150699300_150712342_150712365_150718625_MTMR1 | 150712341-150712365  150718624-150718700  150699202-150699300 | 150712342-150712365 |
| PARD3 | 10 | 34426685-34426731 | 34360259-34377967  34331344-34337365  34269899-34317107 |  | SE_10_-_34374874_34372536_34372498_34360259_PARD3  A5SS_10_-_34337365_34337278_34336243_PARD3 | 34372497-34372536  34374873-34375002  34384128-34384254  34696317-34696419  34470084-34470263  34516978-34517159  34337274-34337426  34337364-34337426  34336198-34336243  34337277-34337426 | 34678861-34679686 |
| NUMA1 | 11 | 72065199-72068231 | 72035975-72080458 |  | AFE_11_-_72041823_72069842_72035975_NUMA1 | 72007188-72008845  72029204-72029290  72035901-72035975 | 72070088-72070093 |
| RABGAP1L | 1 | 174877392-174877605  174877440-174877605  174875546-174875713 | 174252590-174275833  174221164-174241483  174957549-174969277  174811960-174957457 | 174239314-174241483 | SE_1_+_174957549_174957891_174958173_174969277_RABGAP1L | 174877391-174877605  174957890-174958173 |  |
| MDM2 | 12 | 68809024-68809086  68809017-68809086  68809002-68809086  68808282-68808491  68808383-68808491  68808273-68808491  68808464-68808491  68808177-68808491  68808191-68808491 | 68808480-68809208 | 68843832-68843934 | SE_12_+_68808491_68809016_68809086_68809208_MDM2  AFE_12_+_68809086_68808491_68809208_MDM2 | 68816811-68816945  68835828-68835843  68828770-68828931 |  |
| MCM7 | 7 | 100100213-100100757  100100213-100100780  100100213-100100869  100100213-100100473 | 100100093-100101264 | 100100093-100100213 | AFE_7_-_100100213_100101264_100100093_MCM7 | 100099022-100099403  100099022-100099753 |  |
| MEI1 | 22 | 41723943-41724073  41703331-41703454  41718071-41718274  41730521-41730637  41714002-41714075  41729665-41729779  41705504-41705554  41732469-41732603  41731056-41732344  41748107-41748218  41732245-41732344  41745885-41746026  41716041-41716146  41743080-41743194  41744973-41745064  41699503-41699712  41699514-41699712  41781446-41781845  41781399-41781845  41781351-41781845  41781685-41781845 | 41732603-41758365  41781845-41793829  41794477-41795735 | 41701854-41703331  41705554-41712993  41713138-41714002  41724073-41727820  41729779-41730552  41731513-41744330  41744474-41744797 | SE_22_+_41781845_41784339_41784420_41793829_MEI1  SE_22_+_41781845_41784608_41784783_41793829_MEI1 | 41784338-41784420 |  |
| FCGR2B | 1 | 161675257-161675313 | 161671649-161672975  161673229-161673960 | 161674073-161675257 | SE_1_+_161674073_161675257_161675313_161677328_FCGR2B | 161675256-161675313  161673959-161677365 |  |
